# Blood Pressure-Lowering Aspects of Lipid-Lowering and Anti-Diabetic Drugs

**DOI:** 10.3390/ph4010001

**Published:** 2010-12-23

**Authors:** Peter M. Nilsson, Renata Cifkova

**Affiliations:** 1 Department of Clinical Sciences, Lund University Hospital, Malmö, Sweden; 2 Department of Preventive Cardiology, Institute for Clinical and Experimental Medicine, Prague, Czech Republic

**Keywords:** blood pressure, glitazone, metformin, orlistat, rimonabant, sibutramine, sitagliptin, statin

## Abstract

Several studies have shown that blood pressure can be lowered by the use of drugs that are not traditional antihypertensive drugs. This might be of clinical importance when many risk patients are treated by combination drug therapy in order to prevent cardiovascular disease by way of improving the cardiovascular risk factor profile.

## 1. Introduction

Hypertension is a major risk factor for cardiovascular disease on a global scale [1], and the usefulness of blood pressure control has been well proven [2,3]. Traditionally, treatment strategies start with lifestyle interventions followed by antihypertensive drug therapy. Recent studies have, however, shown that a slight blood pressure-lowering effect can also be achieved by other drugs not primarily prescribed for management of hypertension.

These drugs are often prescribed due to comorbidities encountered in hypertensive patients. Examples of such drugs include not only lipid-lowering drugs (statins), anti-diabetic drugs (metformin, glitazones, incretin-enhancing drugs), and weight-reducing drugs (orlistat), but also drugs with temporary effects such as nitrates used as anti-anginal drugs in coronary heart disease. It is useful to know about these effects even if they often seem to be much less pronounced as compared to the effect of the registered and recognised antihypertensive drugs that are discussed in current guidelines for evaluation and treatment of the hypertensive patient [4,5]. Even if the blood pressure reduction is small in itself, it can be influenced by some interesting mechanisms of haemodynamic importance that should be further explored in experimental studies. Examples of such proposed mechanisms include effects related to improved endothelial function, increased insulin sensitivity, or mediated via weight loss.

## 2. Results and Discussion

Table 1 summarizes the data on drug-induced effects on blood pressure levels, based on individual studies and systematic reviews. Some of these are discussed in more detail in the subsections that follow.

### 2.1. Lipid-Lowering Drugs

It has been documented that statin therapy is associated with a few mmHg blood pressure reduction, based on systematic reviews of several intervention trials [6,7]. This could contribute to the beneficial effects of statin therapy on prevention of cardiovascular events [8,9], besides the more important effect on lowering of LDL-cholesterol and a reduction of systemic inflammation, as shown by the effects of rosuvastatin on CRP levels in the JUPITER trial [10]. On the other hand, an increase in glycaemia and newly detected cases of type 2 diabetes has also been noticed. This effect is difficult to explain but could eventually be the result of a decrease in insulin secretion following cholesterol reduction, as has been shown in a laboratory *in vitro* model [11]. If this holds true, the reduction of insulin levels, or even hyperinsulinaemia as a consequence of insulin resistance, could be the link to understand the blood pressure fall, as hyperinsulinaemia has been shown to be associated with elevated blood pressure [12]. Further results from randomised, controlled trials are required to prove or disprove this hypothesis. Nevertheless, statin therapy remains a cornerstone in cardiovascular prevention, irrespective of a slight increase in plasma glucose levels that is overshadowed by the beneficial effects, as outlined above.

### 2.2. Anti-Diabetic Drug Therapy

The aim of using anti-diabetic drugs is foremost to normalize glucose metabolism and to decrease the risk of micro- as well as macrovascular events in patients with either type 1 or type 2 diabetes. Based on intervention studies, this approach has been documented to be successful in both forms of diabetes, even if the therapeutic goal for glycaemic control is still debated. Systematic reviews have recently shown that coronary events, but not stroke events, can be reduced by intensive glycaemic control in patients with type 2 diabetes without a concomitant risk of increasing total mortality rates [13]. One part of this beneficial effect could possibly be attributed to blood pressure lowering. This has been documented in individual studies, as well as in systematic reviews on effects of glitazone (thiazolidinedione) therapy [14,15], but also for metformin (single studies) [16] and exenatide [17], a drug that increases incretin levels of the hormones GLIP-1 and GIP, of importance for glucose metabolism. The effect is linked to a reduction of insulin resistance and improvement of endothelial function (glitazones) or weight loss (exenatide, liraglutide). The effect on blood pressure of metformin therapy is not a consistent finding, but occasionally reported from some smaller studies. The mechanism for the effect, if any, is largely unknown. Another new class of drugs is the so called DPP-4 inhibitors belonging to the incretin class of drugs. It has been shown for both vildagliptin and sitagliptin a reduction of a few mmHg in office systolic blood pressure can be noticed following treatment with these drugs. In addition, it has also been documented that sitagliptin given to non-diabetic, hypertensive subjects might reduce ambulatory systolic blood pressure by 1-2 mmHg in comparison with placebo therapy [18].

On the contrary, it has also been shown that an older type of sulphonylurea drug, chlorpropamide, as used in the UKPDS trial [19], could even increase blood pressure levels, maybe as a consequence of increasing circulating insulin levels resulting in a state characterised by hyperinsulinaemia. What about heamodynamic effects on blood pressure levels following initiation of insulin therapy? No firm data seem to exist but, overall, the blood pressure effect is not of any great importance.

### 2.3. Weight-Losing Drugs

As there exists a linear relationship between increase in body weight and increase of mean blood pressure levels in most subjects [20], as a reflection of insulin resistance and hyperinsulinaemia, it would be expected that weight-losing interventions should also result in blood pressure lowering. This has been noted following treatment with orlistat [21], and even following treatment by rimonabant [22] – a drug that is no longer on the market due to mental side effects. On the other hand, there was some concern from early animal and pre-registration studies that sibutramine, another anti-obesity drug inhibiting norepinephrine reuptake, might induce blood pressure and pulse rate increases. However, the single blind, six-week lead-in period of the SCOUT trial did not show significant overall increases in blood pressure. Approximately only 5% of participants experienced an increase in blood pressure >10 mmHg on two consecutive occasions during the six-week lead-in period. Individuals who had hypertension at baseline showed an appreciable reduction of blood pressure after the six weeks of treatment with sibutramine [23]. The final results of the large-scale SCOUT trial (sibutramine versus placebo) for evaluation of cardiovascular prevention in high-risk individuals [24] were published during 2010. In 10,744 overweight or obese subjects, 55 years of age or older, with preexisting cardiovascular disease, type 2 diabetes mellitus, or both, who were receiving long-term sibutramine treatment, this drug addition led to an increased risk of nonfatal myocardial infarction and nonfatal stroke, but not of cardiovascular death or death from any cause [25]. This caused the withdrawal of sibutramine from the market.

Finally, it should be mentioned that bariatric surgery has been documented to lower blood pressure in the short run (2 years), secondary to weight loss, but with no difference compared to a control group after longer time (8 years) of follow-up, as shown in the Swedish Obese Subject trial [26]. The explanation for this rebound phenomenon in blood pressure is not well understood, but could potentially be explained by the fact that structural vascular changes after many years of obesity are not reversible and may eventually cause an increase in blood pressure following surgical weight loss.

## 3. Conclusions

It has been repeatedly shown that some drugs used for indications other than hypertension are able to affect blood pressure levels, sometimes to a marked degree, but most often just marginally. As cardiovascular risk patients of today are treated more vigorously than previously, the effect of drugs other than conventional antihypertensives on reduction of blood pressure levels could improve blood pressure control. Besides this, the haemodynamic effects shown by some classes of drugs should be further explored in experimental studies to learn more about the potential mechanisms involved. This will benefit the overall understanding of the complexities of blood pressure regulation.

## Figures and Tables

**Table 1:** A summary of drug-induced effects on blood pressure levels, based on individual studies and systematic reviews.

**References**	**Class of drugs**	**Main effect on BP**	**Proposed mechanism**
	Statins	Reduction of 2-4 mmHg	Endothelial function
	Glitazones	Reduction of 2-5 mmHg	Endothelial function, insulin sensitivity
	Exenatide, Liraglutide	Reduction of 2-3 mmHg	Weight loss
	DPP-4 inhibitors	Reduction of 2 mmHg	Unknown
	Metformin	Variable	Unknown
	Orlistat	Reduction of 2-5 mmHg	Weight loss
	Sibutramine	Variable	Influence on sympathetic nerve activity
	Sympathomimetics	Hypertensive crisis	Norepinephrine increase
	Oral contraceptives	Mild BP elevation in most women and overt hypertension in about 5%	Increase in body weight, plasma and systolic volume, RAAS activation, insulin resistance induction

RAAS; renin-angiotensin-aldosterone system.
